# Comparative Genomic Hybridization Analysis Shows Different Epidemiology of Chromosomal and Plasmid-Borne *cpe*-Carrying *Clostridium perfringens* Type A

**DOI:** 10.1371/journal.pone.0046162

**Published:** 2012-10-19

**Authors:** Päivi Lahti, Miia Lindström, Panu Somervuo, Annamari Heikinheimo, Hannu Korkeala

**Affiliations:** Department of Food Hygiene and Environmental Health, Faculty of Veterinary Medicine, University of Helsinki, Helsinki, Finland; Aarhus University, Denmark

## Abstract

*Clostridium perfringens*, one of the most common causes of food poisonings, can carry the enterotoxin gene, *cpe*, in its chromosome or on a plasmid. *C. perfringens* food poisonings are more frequently caused by the chromosomal *cpe*-carrying strains, while the plasmid-borne *cpe*-positive genotypes are more commonly found in the human feces and environmental samples. Different tolerance to food processing conditions by the plasmid-borne and chromosomal *cpe*-carrying strains has been reported, but the reservoirs and contamination routes of enterotoxin-producing *C. perfringens* remain unknown. A comparative genomic hybridization (CGH) analysis with a DNA microarray based on three *C. perfringens* type A genomes was conducted to shed light on the epidemiology of *C. perfringens* food poisonings caused by plasmid-borne and chromosomal *cpe*-carrying strains by comparing chromosomal and plasmid-borne *cpe*-positive and *cpe*-negative *C. perfringens* isolates from human, animal, environmental, and food samples. The chromosomal and plasmid-borne *cpe*-positive *C. perfringens* genotypes formed two distinct clusters. Variable genes were involved with *myo*-inositol, ethanolamine and cellobiose metabolism, suggesting a new epidemiological model for *C. perfringens* food poisonings. The CGH results were complemented with growth studies, which demonstrated different *myo*-inositol, ethanolamine, and cellobiose metabolism between the chromosomal and plasmid-borne *cpe*-carrying strains. These findings support a ubiquitous occurrence of the plasmid-borne *cpe*-positive strains and their adaptation to the mammalian intestine, whereas the chromosomal *cpe*-positive strains appear to have a narrow niche in environments containing degrading plant material. Thus the epidemiology of the food poisonings caused by two populations appears different, the plasmid-borne cpe-positive strains probably contaminating foods via humans and the chromosomal strains being connected to plant material.

## Introduction

Enterotoxin gene-carrying (*cpe*-positive) *Clostridium perfringens* type A is one of the most common causes of food poisoning in the industrialized world, and the third leading cause of food poisoning in USA [1]. Limited knowledge of the reservoirs and the contamination routes of *cpe*-positive *C. perfringens* complicates the prevention of *C. perfringens* food poisonings.

*C. perfringens* is an anaerobic ubiquitous spore-forming bacterium, frequently present in the normal intestinal microbiota of humans and animals. *C. perfringens* strains are classified into different types (A–E) based on their expression of alpha, beta, epsilon, and iota toxins [2]. *C. perfringens* can cause several diseases in humans and animals due to the variety of toxins it produces.

Fewer than 5% of *C. perfringens* type A strains carry the enterotoxin gene *cpe* [3]. The *cpe* can be located in the bacterial chromosome or on a large plasmid [4]–[6]. The chromosomal *cpe* is flanked by an insertion sequence (IS) element IS*1470* (*cpe*-genotype IS*1470*) [4], whereas the plasmid-borne *cpe* is flanked by either the IS*1470*-like or IS*1151* element (*cpe*-genotypes IS*1470*-like or IS*1151*) [6], [5]. Until recently, only the chromosomal *cpe*-carrying strains were associated with food poisonings [7]. This was explained by their better tolerance to heating, low temperatures, and preservatives than that of the plasmid-borne *cpe*-carrying strains [8], [9]. However, also the plasmid-borne genotypes were found to cause food poisonings [10]–[12] and the *cpe* was carried on a plasmid in 25% of food poisoning outbreaks investigated in Finland and Germany [12].

Both chromosomal and plasmid-borne c*pe*-positive *C. perfringens* genotypes were found in retail meat products [13], [14], but the contamination route remains unknown. The contamination of meat by the intestinal contents of slaughtered animals has been suggested to serve as the main source of *cpe*-positive *C. perfringens* [15]. However, no successful isolations of *cpe*-positive *C. perfringens* strains have been reported from healthy production animals [16]–[18]; thus, the role of animals as the main reservoir of *cpe*-positive *C. perfringens* has been questioned [18].

Humans are a rich reservoir for plasmid-borne *cpe*-carrying strains [19], [20] and were thus suggested to introduce a contamination risk into foods through handling [18]. However, only a few chromosomal strains were found in human feces [19]. Plasmid-borne *cpe*-positive strains were also detected in soil and sediments [21], [22]. For better prevention of *C. perfringens* food poisonings, the reservoirs of the *cpe*-positive *C. perfringens* strains and the potentially different epidemiology of *C. perfringens* type A food poisonings caused by the chromosomal and plasmid-borne *cpe*-carrying strains need to be elucidated.

Comparative genomic hybridization (CGH) with DNA microarrays was performed to shed light on the epidemiology of the chromosomal and plasmid-borne *cpe*-carrying and *cpe*-negative *C. perfringens* type A strains of food, human, or animal origin. The results of the CGH analysis were complemented with growth studies, which demonstrated different metabolism between the chromosomal and plasmid-borne *cpe*-carrying strains. The results suggest different epidemiology of the *cpe*-positive *C. perfringens* groups, which is relevant when designing prevention of *C. perfringens* food poisonings.

## Results

To assess genetic relatedness and possible metabolic differences between the chromosomal and plasmid-borne *cpe*-positive and *cpe*-negative *C. perfringens* strains, a DNA microarray was designed based on three sequenced *Clostridium perfringens* genomes ATCC13124, strain 13 and SM101. A wide collection of *C. perfringens* strains (n = 83) from food and feces associated with food poisonings, feces of healthy humans, feces of healthy production animals, soil and sludge, were studied (Table S1). The strains represented different *cpe*-positive genotypes and *cpe*-negative strains, the latter including the reference strains ATCC13124 and 13 which were used as positive controls. A two-color labeling system was used and the differently labeled DNA sample pairs to be hybridized into one of the eight subarrays on each array slide were randomly selected. Reproducibility of the hybridizations was controlled by hybridizing 20 samples in duplicate and the control strains in quadruplicate. The DNA samples of the reference strains 13 and ATCC 13124 hybridized 99.9% with their own gene probes. The putative metabolic differences suggested by the CGH analysis were further confirmed by metabolic tests using minimal growth medium. All strains tested grew in the minimal medium with glucose as the sole carbon source and failed to grow in minimal medium without any source of carbon, demonstrating that the medium supported the growth of *C. perfringens*.

**Figure 1:**
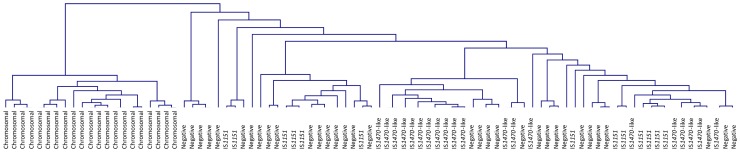
Chromosomal *cpe*-carrying *C. perfringens* strains clustered separately from the plasmid-borne *cpe*-carrying and *cpe*-negative strains. The figure was constructed using the MEV software [28].

The 54 *cpe*-positive *C. perfringens* type A strains formed two distinct clusters, one consisting of the chromosomal *cpe-*carrying genotypes and the other of the plasmid-borne *cpe*-carrying genotypes (Figure S1). The similarity between strains, based on Pearson's correlation on a scale from −1 to 1, was 0.85 in the chromosomal *cpe* group and 0.76 in the plasmid-borne *cpe* group. The similarity between the two groups of *cpe*-positive *C. perfringens* strains was 0.59 (Figure 1). When the 29 *cpe*-negative strains were included in the analysis, the chromosomal strains still clustered separately, and the plasmid-borne *cpe*-carrying strains and the *cpe-*negative strains were evenly distributed in the other cluster. The chromosomal cluster was homogeneous, whereas the cluster consisting of plasmid-borne *cpe*-carrying or plasmid-borne *cpe*-carrying and *cpe*-negative strains was more heterogeneous (Figure 1).

In general, the plasmid-borne *cpe*-carrying strains shared more CDSs (75,6%–87,4%) with the *cpe*-negative reference strains ATCC 13124 and 13 than with the chromosomal *cpe*-carrying reference strain SM101 (71,8%–84,4%) (Table 1). By contrast, the chromosomal *cpe*-positive *C. perfringens* strains shared more CDSs with the reference strain SM101 (86,2%-94,9%) than with the two other reference strains (63,8%–81,4%) (Table 1). Altogether 372 CDSs were exclusively present in the plasmid-borne *cpe*-positive strains, and 242 CDSs were exclusively present in the chromosomal *cpe*-carrying strains.

**Table 1:** Minimum and maximum percentage of CDSs in the three reference strains (SM101, ATCC13124, and 13) carried by chromosomal and plasmid-borne *cpe*-carrying and *cpe*-negative *C. perfringens* strains.

*cpe* location	SM101		ATCC13124		Strain 13	
	min	max	min	max	min	max
Chromosomal	86.2	94.9	70.7	81.4	63.8	74.8
Plasmid-borne	71.8	84.4	82.8	91.5	75.6	87.4
*cpe*-negative	73.4	85.3	80.4	99.9	74.5	99.0

When the CDSs of the reference strains were divided into functional groups based on J. Craig Venter Institute Comprehensive Microbial Resource (CMR) annotations, the plasmid-borne *cpe*-positive strains carried more CDSs than chromosomal strains of all except two functional groups: transposable elements, and protein synthesis and electron transport (Table S2). Marked differences were present in the numbers of CDSs without specific annotation (Table S2).

The major differences between the chromosomal and plasmid-borne *cpe*-carrying strains were in the presence of the operons related to *myo*-inositole and ethanolamine utilization; a gene cluster encoding phosphotransferases and beta-glucanases, including laminarinase and cellobiose phosphotransferase; and a gene cluster encoding biotin synthesis.

**Figure 2:**
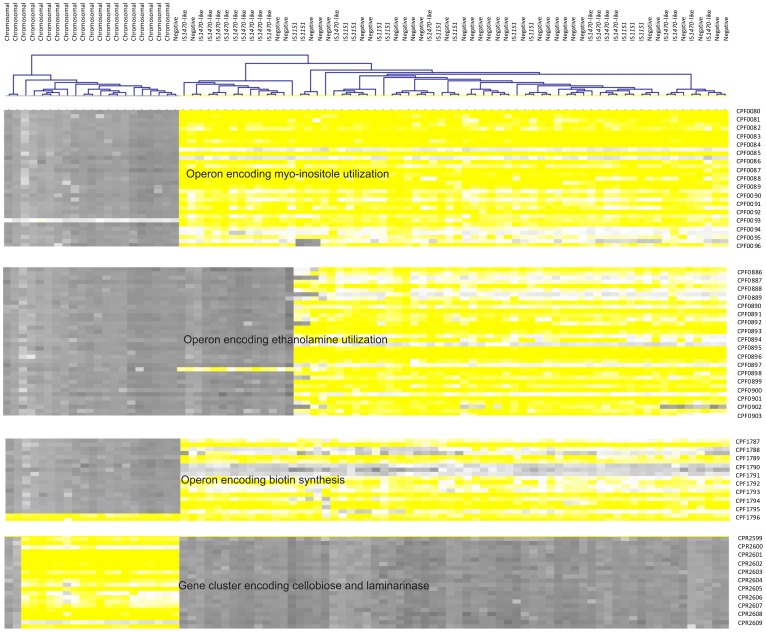
Genes differentiating the chromosomal *cpe*-carrying *C. perfringens* strains from the plasmid-borne *cpe*-carrying and *cpe*-negative strains. The figure was constructed using the MEV software [28].

All plasmid-borne *cpe*-carrying strains carried the *myo*-inositol operon, whereas all chromosomal *cpe*-positive strains lacked this operon (Figure 2, Table S4). Accordingly, all tested plasmid-borne *cpe*-positive *C. perfringens* strains and none of the tested chromosomal *cpe*-positive strains utilized myo-inositol (Table S3). In the reference strain ATCC13124, the *myo*-inositol operon is located in the chromosome and consists of 13 CDSs (locus CPF0079–CPF0092). *iolR* upstream of the cluster is predicted to encode a divergent regulator.

All the 21 chromosomal *cpe*-carrying strains lacked the operon predicted to encode ethanolamine utilization, whereas 23 of the 33 plasmid-borne *cpe*-carrying strains, including all strains representing genotype IS*1151*-*cpe* and ten of 20 strains representing genotype IS*1470*-like-*cpe*, had this operon (Figure 2, Table S4). Again, the result was verified by all tested plasmid-borne *cpe*-positive *C. perfringens* strains and none of the tested chromosomal *cpe*-positive strains utilizing myo-inositol (Table S3). The ethanolamine utilization operon is found in the genomes of the reference strains ATCC13124 and 13, and it contains 17 CDSs.

Nearly all (19 of 21) chromosomal *cpe*-carrying strains had a gene cluster predicted to encode cellobiose phosphotransferase, laminarinase, and beta-glucanases, whereas all plasmid-borne *cpe*-carrying strains lacked this gene cluster. In support of the CGH data, all the chromosomal *cpe*-positive strains tested utilized cellobiose as the only carbon source (Table S3). Of the 12 plasmid-borne *cpe*-positive strains that lacked this gene cluster, nine failed to grow in minimal medium with cellobiose as the sole carbon source, and three grew in the minimal medium with cellobiose despite lacking the gene cluster. The gene cluster predicted to encode phosphotransferases and beta-glucanases contains 11 CDSs and is located in the chromosome of the *cpe*-positive SM101 (locus CPR2599–CPR2609) (Figure 2, Table S4). Moreover, all chromosomal *cpe*-positive *C. perfringens* strains lacked a gene cluster containing nine CDSs, of which *bioB* and *bioD* encode biotin synthesis (locus CPF1787–1795 in ATCC 13124). All 83 strains carried *bioY* (CPF1796), predicted to encode biotin intake.

The genomic content of the *cpe*-negative strains resembled that of the the plasmid-borne *cpe*-gene carrying strains. All *cpe*-negative strains carried an operon for *myo*-inositol utilization, most (26 of 29) carried the operon encoding ethanolamine utilization, all carried the gene cluster encoding biotin synthesis, and all lacked the gene cluster encoding phosphotransferases and beta-glucanases.

## Discussion

The chromosomal and the plasmid-borne *cpe*-carrying *C. perfringens* type A strains differed in their gene composition and clustered separately in the CGH analysis. The microarray results were confirmed by functional metabolic studies. The main differences were related to genes involved in the utilization of *myo*-inositole, ethanolamine, and cellobiose, and the synthesis of biotin. Accordingly, different ability of the chromosomal and plasmid-borne *cpe*-positive strains to utilize *myo*-inositole, ethanolamine, and cellobiose as the only source of energy was demonstrated. This suggests that the chromosomal and plasmid-borne *cpe*-carrying *C. perfringens* strains are differently adapted to various environments, and thus, the epidemiology of *C. perfringens* food poisoning caused by the two strain populations may be different.

The plasmid-borne *cpe*-carrying and the *cpe*-negative strains formed a heterogeneous group, with some plasmid-borne *cpe*-carrying and *cpe*-negative strains being very similar. This supports horizontal transfer of the *cpe* plasmid between *C. perfringens* strains, as proposed in previous studies [19], [23], [24].

The chromosomal *cpe*-positive strains formed a homogeneous cluster, which is in agreement with an earlier study using multi-locus sequence typing [25]. It seems plausible that the chromosomal *cpe*-positive strains have diverged from the remaining *C. perfringens* population, which is ubiquitous in nature and consists of a heterogeneous group of *cpe*-negative but also plasmid-borne *cpe*-carrying strains. Although the chromosomal *cpe*-carrying strains appear to better survive in certain extreme conditions [8], [9], the present results suggested that the plasmid-borne *cpe*-carrying and *cpe*-negative strains have specific properties by which they are better adapted to diverse environments than the chromosomal *cpe*-carrying strains.

The property of both the plasmid-borne *cpe*-positive and *cpe*-negative strains, utilizing *myo*-inositol, suggests that these strains are similarly adapted to multiple habitats. Apart from being abundant in the soil and environment, *myo*-inositol is a component of the eukaryotic cell wall and has been reported to be used by *C. perfringens* as an alternative carbon source in the absence of glucose [26]. Several microorganisms inhabiting the soil can utilize *myo*-inositol [26]. The absence of this operon in all chromosomal *cpe*-carrying strains may limit their ubiquitous occurrence considered typical for *C. perfringens*, which suggests that the chromosomal *cpe*-carrying strains have their own, an as-yet unidentified narrow niche in the environment.

Since ethanolamine is abundant in the human intestine [27], the presence of the operon encoding ethanolamine utilization in 70% of the plasmid-borne *cpe*-carrying strains and in 90% of the *cpe*-negative strains probably provides an advantage for survival and colonization of the intestine for these strains [19], [27]. The clostridial ethanolamine operon resembles that of *Enterobacteriacae* [28], among which ethanolamine utilization is common. Due to their ability to utilize ethanolamine, the plasmid-borne *cpe*-carrying strains could be adapted to the intestinal environment, unlike the chromosomal *cpe*-carrying strains, that lacked the ethanolamine utilization operon.

Biotin is involved in the central pathways of cell metabolism, and bacteria unable to synthesize biotin need to acquire it from external sources [29]. The lack of this gene cluster in the chromosomal *cpe*-positive strains may indicate that the habitat of these strains is rich in free biotin.

The ability of the chromosomal *cpe*-positive *C. perfringens* strains to utilize cellobiose obtained by enzymatic or acidic hydrolysis of cellulose and laminarin common in plant cell walls may indicate that these polysaccharides are available in the yet unknown habitat of the chromosomal *cpe*-positive strains. Despite lacking the gene cluster predicted to encode utilization of cellobiose, three plasmid-borne *cpe*-carrying strains utilized cellobiose, which may indicate that cellobiose utilization is encoded by multiple loci, not necessarily represented in our microarrays.

Based on the currently available genome sequences, we expect the *cpe*-positive *C. perfringens* strain population to contain hundreds of genes not present in the reference genomes and thus not represented on the microarrays. For example, majority of the 73 and 62 genes of the *cpe*-containing plasmids pCPF5603 and pCPF4969 [30], respectively, are specific to plasmids, since they share only 10 and 7 genes with SM101 according to BLAST. Therefore one should bear in mind that the differential gene pool observed in this study is likely to be larger and warrants future study.

The chromosomal *cpe*-carrying *C. perfringens* strains seem unable to utilize *myo*-inositol or ethanolamine or to synthesize biotin, which are important for soil and intestinal bacteria competing in complex environments. The majority of the chromosomal *cpe*-carrying strains in this study also lacked the fucose and sialidase encoding genes, which further diminishes the territory of the chromosomal *cpe*-positive strains [31]. Presumably, the chromosomal *cpe*-positive strains are not ubiquitous and soil or intestines are not the habitat of these strains, although the chromosomal strains may compensate for some of the aforementioned deficiencies by producing toxins or by acquiring appropriate genes from the environment. This is supported by the presence of many IS-elements suggestive of gene transfer [31].

In light of our results, the habitat of the chromosomal *cpe*-carrying *C. perfringens* strains appears to be rich in biotin, and the ability to utilize cellobiose and laminarin may be beneficial. Cellobiose, laminarin, and biotin are available in environments where bacteria decompose plant material, such as composts. In composts, the temperature may be high, allowing only the most heat-tolerant strains, such as the chromosomal *cpe*-carrying strains, to survive. Access of the spores of chromosomal *cpe*-carrying *C. perfringens* to the food chain via the compost soil on the surface of vegetables should be investigated.

Other environments rich in biotin and cellobiose include sewage and sludge [32], where the chromosomal *cpe*-carrying *C. perfringens* strains may end up via the excretions of food-poisoning patients. The heat-resistant spores of the chromosomal *cpe*-positive strains could also tolerate heat treatments and drying [8], [9], which are usually included in the waste water treatment procedures. Thus, the role of sewage and sludge as a reservoir of chromosomal *cpe*-positive *C. perfringens* should be addressed, as the spores surviving the waste water treatment procedures may return to the food chain via sludge used as fertilizer.

In conclusion, the results suggest the plasmid-borne *cpe*-carrying strains and *cpe*-negative strains to be ubiquitous and adaptated to the mammalian intestine. By contrast, the chromosomal *cpe*-carrying strains appear to have a narrow niche in environments containing degrading plant material. Thus, the plasmid-borne *cpe*-carrying strains are proposed to contaminate foods by human due to poor hygiene, whereas the chromosomal *cpe*-carrying strains could spread to the food chain through ingredients of plant origin. Further research is needed to elucidate the habitat of these strains.

## Materials and Methods

### Bacterial strains

A total of 83 *C. perfringens* type A strains isolated from foods (n = 19) and feces (n = 9) associated with food poisonings, feces of healthy (n = 21) and ill (n = 6) people, feces of healthy production animals (pigs n = 7, cattle n = 5, broiler chickens n = 5), soil (n = 5), and sludge (n = 6) during 1986–2007 included 54 *cpe*-positive strains and 29 *cpe*-negative strains (Table S1). Of the *cpe*-positive strains, a chromosomal *cpe* was carried by 21 strains while 33 carried the *cpe* on a plasmid. Of the plasmid-borne *cpe*-carrying strains, 20 represented genotype IS*1470*-like and 13 represented genotype IS*1151*. The *cpe*-negative *C. perfringens* strains ATCC 13124 and 13, and the chromosomal *cpe-*positive strain SM101 were used as hybridization references. Genomic DNA of all strains was isolated as described by Keto-Timonen et al. (2005) [33].

### DNA microarrays

The DNA microarrays, based on the genomes of *C. perfringens* type A strains 13 [34], ATCC13124, and SM101 [31], contained two 60-mer probes for all protein coding sequences (CDSs) annotated in the three genomes. The probes were designed using the OligoArray2.1 software [35]. Upon the probe design, OligoArray2.1 software utilizes the BLAST algorithm for checking the specificity of a probe. There are 2170 conserved genes (core genome) in all the three reference genomes and over 2000 strain-specific genes or genes present only in two of the reference strains. First, probe design was done for each individual strain, and then the results were combined. Up to five candidate probes were designed for each CDS. Finally a maximum of two probes per CDS were selected, not accepting any duplicate probes. Moreover, four probes for each IS element (IS*1469*, IS*1470*, IS*1470*-like, and IS*1151*) [6], [4], [5] were included for genotyping of *cpe*-positive *C. perfringens*. Each of the eight sub-arrays of Agilent 8*15K custom arrays (Agilent, Santa Clara, CA, USA) contained an equal set of 15 744 probes.

### Hybridization and washes

A total of 0.5 mg of genomic DNA from each *C. perfringens* strain was fluorescently labeled using the BioPrime labeling kit (Invitrogen, Carlsbad, CA, USA). The 26-µl labeling reaction contained 11.5 µl of diluted DNA, 10 µl of random octamer primers (Invitrogen), 2.5 µl of 10× dCTP Nucleotide mix (Invitrogen), 1.5 µl of either Cy3 or Cy5-dCTP (GE Healthcare, Buckinghamshire, UK), and 0.5 µl of Exo-Klenow fragment solution (Invitrogen). The reactions were incubated at 37°C for 2 hours and stopped by adding 2.5 µl of stop buffer (EDTA, Invitrogen). For each hybridization, one Cy3-labeled and one Cy5-labeled DNA sample were combined; thus two samples were hybridized on each subarray and 16 samples on each array slide. The mixture was purified with a DNA purification kit (QIAquick PCR Purification Kit, Qiagen, Hilden, Germany) according to the manufacturer's instructions. The concentration of DNA and the incorporation of the dye were checked with the Nanodrop device (Nanodrop Technologies, Wilmington, MA, USA) before and after labeling. A volume of 2.2 µl salmon sperm DNA (1 mg/ml) was added to 17.8 µl of labeled combined sample solution, and the mixture was heated at 95°C for 2 minutes for denaturation. A volume of 5 µl of 10× blocking agent (Agilent) and 25 µl 2xGE (HI-RPI) hybridization buffer (Agilent) were added. A total of 45 µl of the solution was hybridized to each microarray at 65°C for 16 hours. The arrays were washed for 2×1 minute with Wash Buffer 1 (Agilent) and for 1 minute with Wash Buffer 2 (Agilent), pre-warmed to 37°C.

### Scanning, image processing and data analysis

The slides were scanned (Axon GenePix Autoloader 4200 AL, Westburg, Leusden, The Netherlands) using 5 µm pixel resolution. Image processing was performed with the GenePix Pro 6.0 software. All hybridizations were normalized to the reference strains after background correction. Since the probes were designed based on three genomes, the location of the main mode of log_2_-ratio distribution was calculated between the hybridized strain and all reference strains, and the median value was used for normalization.

The distribution of logarithmic signal intensities formed two clear peaks in each hybridization. A threshold was set between the peaks based on replicated hybridizations of the two reference strains ATCC13124 and 13; signal intensities from the probes designed for the reference strain were above the threshold. The selected threshold divided the probes into two groups: The peak with greater values corresponded to probes with specific hybridization and genes predicted to be present, and the peak below the threshold corresponded to probes predicting a gene to be absent/divergent or yiedling unspecific hybridization and. The data analysis was done using the R software [36], and visualization and clustering were conducted using MEV [37]. The data discussed in this publication are compliant with the MIAME guidelines and were deposited in NCBI's Gene Expression Omnibus and are accessible through GEO Series accession number GSE30954 (http://www.ncbi.nlm.nih.gov/geo/query/acc.cgi?acc=GSE30954).

To validate the DNA microarray results, the intensity of the IS element, *plc* (encoding the alpha toxin), and the *cpe* probe spots was compared with results of PCR assays showing the IS elements downstream of *cpe* [4], [5], [23] and the presence of *plc* and *cpe* [38]. The signal intensity values of all validated probe spots were in concordance with the PCR results.

### Myo-inositol, cellobiose, and ethanolamine utilization of the *cpe*-positive *C. perfringens* strains

The minimal medium was prepared according to Sebald and Costilow (1975) [39]. The growth of 10, 10, and 8 chromosomal, and 11, 12, and 7 plasmid-borne *cpe*-carrying *C. perfringens* strains was examined in the minimal medium using *myo*-inositol, cellobiose, and ethanolamine, respectively, as the sole source of energy. For controls, the growth of each strain was also examined in minimal medium with glucose and in a plain minimal medium. In brief, 25 µl of a 5×10^4^ cfu/ml cell suspension of each strain was inoculated into 2.5 ml of fresh minimal medium containing 1% of either *myo*-inositol, cellobiose, or ethanolamine and incubated at 37°C overnight under anaerobic conditions. Growth in the presence of *myo*-inositol and cellobiose was studied in an automated turbidity reader (Bioscreen C Microbiology Reader, Growth Curves, Helsinki, Finland). To demonstrate ethanolamine utilization, 0.05% adenocylcobalamine, which is considered essential for ethanolamine consumption [27], was added to the media together with bromothymol blue as an indicator. Growth in the presence of ethanolamine was studied in 10-ml aliquots. A change of the indicator colour suggested ethanolamine utilization.

## Supporting Information

Figure S1 **Similarity between the strains in the clusters of chromosomal and plasmid-borne** ***cpe*** **-carrying** ***C. perfringens*** **strains.** The similarity between the chromosomal *cpe*-carrying strains is 0.85 (Pearson's correlation) The similarity between the plasmid-borne *cpe*-carrying strains (IS*1470*-like and IS*1151*) is 0.76, and the similarity between the two clusters is 0.59.
(TIF)

Table S1 Characterization of *Clostridium perfringens* type A strains isolated from various sources.
(RTF)

Table S2 Variable CDSs (probes) in chromosomal *cpe*-carrying *C. perfringens* strains related to plasmid-borne *cpe*-carrying strains.
(RTF)

Table S3 Utilization of *myo*-inositole, ethanolamine, and cellobiose of chromosomal and plasmid-borne *cpe*-carrying *C. perfringens* strains.
(RTF)

Table S4 The presence (+) and absence (−) of operons and gene clusters encoding the metabolic traits differentiating between the chromosomal and plasmid-borne *cpe*-carrying *C. perfringens* strains.
(RTF)
