# Supplementary figures and images for: Comparative Genomic Hybridization Analysis Shows Different Epidemiology of Chromosomal and Plasmid-Borne cpe-Carrying Clostridium perfringens Type A
Source: PLoS One. 2012 Oct 19;7(10):e46162. doi: 10.1371/journal.pone.0046162 (PMC3477167; doi:10.1371/journal.pone.0046162)

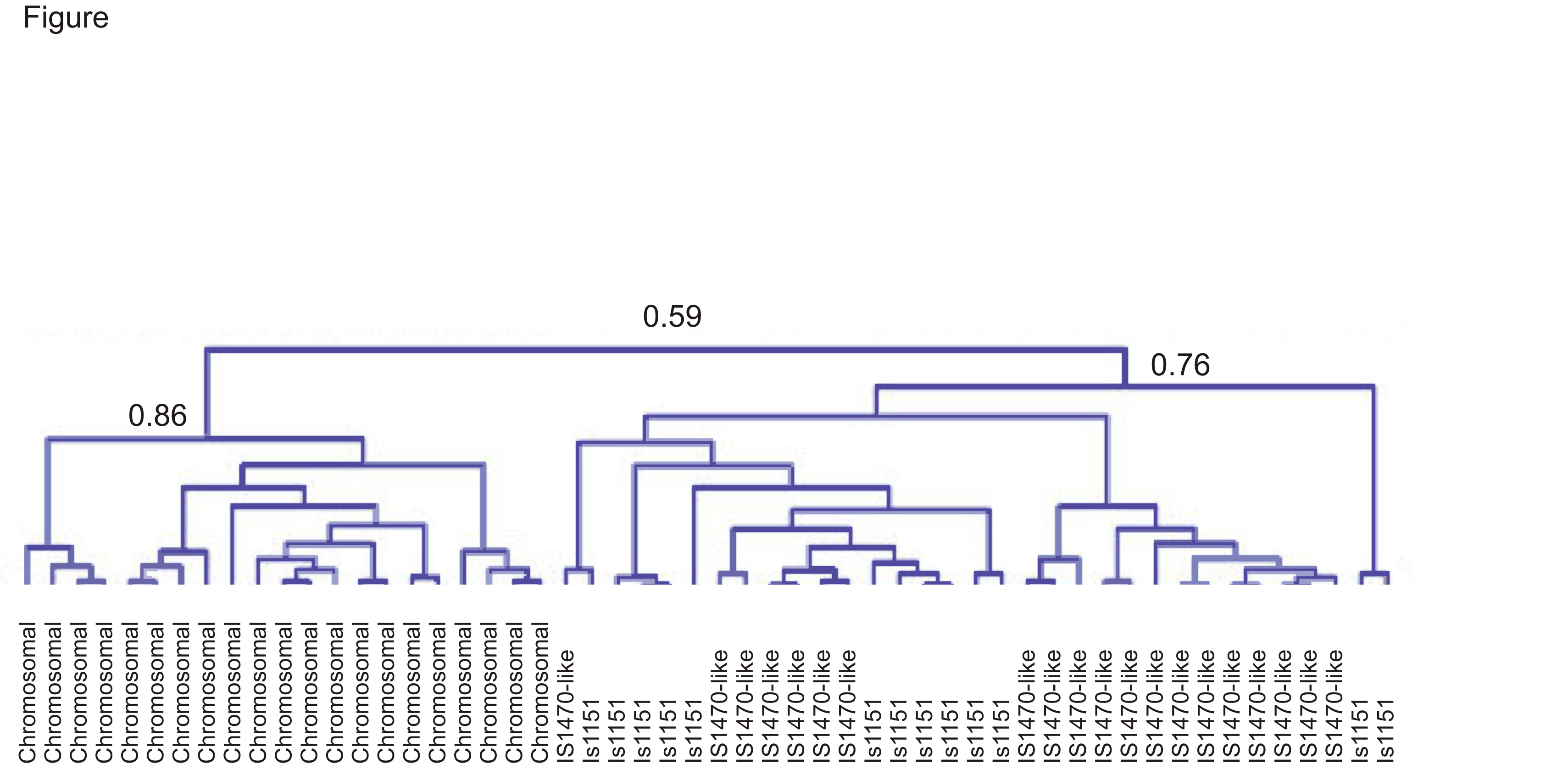

Supplement: Figure S1 — Similarity between the strains in the clusters of chromosomal and plasmid-borne cpe -carrying C. perfringens strains. The similarity between the chromosomal cpe-carrying strains is 0.85 (Pearson's correlation) The similarity between the plasmid-borne cpe-carrying strains (IS1470-like and IS1151) is 0.76, and the similarity between the two clusters is 0.59. (TIF) [file pone.0046162.s001.tif]
