# Supplementary material for: Comparative Genomic Hybridization Analysis Shows Different Epidemiology of Chromosomal and Plasmid-Borne cpe-Carrying Clostridium perfringens Type A
Source: PLoS One. 2012 Oct 19;7(10):e46162. doi: 10.1371/journal.pone.0046162 (PMC3477167; doi:10.1371/journal.pone.0046162)
Supplement: Table S1 — Characterization of Clostridium perfringens type A strains isolated from various sources. (RTF) [file pone.0046162.s002.rtf]

Table S1. Characterization of Clostridium perfringens type A strains isolated from various sources.
	

Strain	
cpe-genotypea	
Chromosomal cpe C/
Plasmid-borne cpe Pa
	
Originb	

CPI76K-4
	
IS 1470	
C	
Human feces	
CPLi7B3
	IS 1470	C	Sludge	
CPR13
	IS 1470	C	Food associated to FP	
CPR14
	IS 1470	C	Food associated to FP	
CPR15
	IS 1470	C	Food associated to FP	
CPR17
	IS 1470	C	Food associated to FP	
CPR18
	IS 1470	C	Food associated to FP	
CPR19
	IS 1470	C	Food associated to FP	
CPR20
	IS 1470	C	Food associated to FP	
1293/86
	IS 1470	C	Food associated to FP	
20/85
	IS1470	C	Food associated to FP	
26/86
	IS1470	C	Food associated to FP	
310/85
	IS 1470	C	Food associated to FP	
318/86
	IS1470	C	Feces associated to FP	
346/91
	IS1470	C	Feces associated to FP	
497/86
	IS1470	C	Feces associated to FP	
834/87
	IS 1470	C	Food associated to FP	
945/85
	IS 1470	C	Food associated to FP	
948/85
	IS 1470	C	Food associated to FP	
954/84
	IS1470	C	Feces associated to FP	
955/85
	IS1470	C	Feces associated to FP	
AAD1863c
	IS1470-like	P	Human feces, associated to AAD
	
AAD1900a
	IS1470-like	P	Human feces, associated to AAD
	
SIDS14os
	IS1470-like	P	Human intestinal content, associated to SIDS
	
CPI 18-1b
	IS1470-like	P	Human feces	
CPI4I 18-2
	IS1470-like	P	Human feces	
CPI 18-4
	IS1470-like	P	Human feces	
CPI 39-1a
	IS1470-like	P	Human feces	
CPI 57K-1
	IS1470-like	P	Human feces	
CPI 57K-4
	IS1470-like	P	Human feces	
CPI 75K-4
	IS1470-like	P	Human feces	
CPI 75-4
	IS1470-like	P	Human feces	
CPLi 2-1
	IS1470-like	P	Sludge	
CPLi 6-1
	IS1470-like	P	Sludge	
CPM 77b
	IS1470-like	P	Soil	
CPM 77e
	IS1470-like	P	Soil	
CPR11
	IS1470-like	P	Food associated to FP	
CPR12
	IS1470-like	P	Food associated to FP	
CPR16
	IS1470-like	P	Food associated to FP	
149/92
	IS1470-like	P	Feces associated to FP	
1533/86
	IS1470-like	P	Feces associated to FP	
AAD 1527a
	IS1151	P	Human feces, associated to AAD
	
AAD 1930/04a
	IS1151	P	Human feces, associated to AAD	
CPI 103K-3
	IS1151	P	Human feces	
CPI 26k-r2
	IS1151	P	Human feces	
CPI 53k-r1
	IS1151	P	Human feces	
CPI 63K-r5
	IS1151	P	Human feces	
CPI 75K-2a
	IS1151	P	Human feces	
CPI 75K-3
	IS1151	P	Human feces	
CPI 75-1
	IS1151	P	Human feces	
CPLi3-1
	IS1151	P	Sludge	
CPLi4-1
	IS1151	P	Sludge	
721/84
	IS1151	P	Food associated to FP	
SIDS 15ps
	IS1151	P	Human intestinal content, associated to SIDS
	
CPB(bp70-1/1)
	-	-	Broiler feces	
CPB (bp70-2/4)
	-	-	Broiler feces	
CPB (bp70-3/4)
	-	-	Broiler feces	
CPB (bp70-5/1)
	-	-	Broiler feces	
CPB (bp70-6/4)
	-	-	Broiler feces	
CPI 18-6
	-	-	Human feces	
CPI 39K-1a
	-	-	Human feces	
CPI 39k-2a
	-	-	Human feces	
CPI 57-2
	-	-	Human feces	
CPLi8B2
	-	-	Sludge	
CPM 4b
	-	-	Soil	
CPM 42a
	-	-	Soil	
CPM 7a
	-	-	Soil	
CPN 16b
	-	-	Cattle feces	
CPN 17a
	-	-	Cattle feces	
CPN 29a
	-	-	Cattle feces	
CPN 35d
	-	-	Cattle feces	
CPN 7c
	-	-	Cattle feces	
CPS 12KM
	-	-	Pig feces	
CPS 131-3a
	-	-	Pig feces	
CPS 2a
	-	-	Pig feces	
CPS 217b
	-	-	Pig feces	
CPS 253-c2a
	-	-	Pig feces	
CPS 30 c
	-	-	Pig feces	
CPS 50a
	-	-	Pig feces	
D9033/97
	-	-	Human feces	
148/92
	-	-	Feces associated to FP	
1534/86
	-	-	Feces associated to FP	
344/91
	-	-	Food associated to FP	
a -, strain is cpe-negative
b FP, food poisoning; AAD, antibiotic associated diarrhea; SIDS, sudden infant death syndrome
