# Supplementary material for: Comparative Genomic Hybridization Analysis Shows Different Epidemiology of Chromosomal and Plasmid-Borne cpe-Carrying Clostridium perfringens Type A
Source: PLoS One. 2012 Oct 19;7(10):e46162. doi: 10.1371/journal.pone.0046162 (PMC3477167; doi:10.1371/journal.pone.0046162)
Supplement: Table S2 — Variable CDSs (probes) in chromosomal cpe-carrying C. perfringens strains related to plasmid-borne cpe-carrying strains. (RTF) [file pone.0046162.s003.rtf]

Table S2. Variable CDSs  (probes) in chromosomal cpe-carrying C. perfringens strains related to plasmid-borne cpe-carrying strains. The CDSs functional classification is according to Myers et al. (2006), obtained from the database of  the J. Craig Venter Institute. Only significant (p<0.05) variability in medians of the CDSs of the functional class as tested with Fisher's exact test is shown.
	
CDS (probes) functional class
	Number of the CDSs (probes) in microarray	Median (%) of the number of CDSs (probes) carried by the chromosomal strains
	Median (%) of the number of CDSs (probes) carried by the plasmid-borne  strains	
Energy metabolism				
Aerobic, Anaerobic, Other
	22	13 (59.1)	21 (95.5)	
Energy metabolism, transport and binding proteins
Amino acids and amines
Cations and iron carrying compounds
Unknown substrate
				
	298	242 (81.2)	284 (95.3)	
	243	190 (78.2)	225 (92.6)	
	302	228 (75.5)	266 (88.1)	
Biosynthesis of cofactors
Biotin
				
	18	10 (55.6)	18 (100)	
Signal transduction
Carbohydrates, organic alcohols, and acids, PTS
DNA interactions, Two-component systems
				
	66	36 (54.5)	55 (83.3)	
	89	68 (76.4)	83 (93.3)	
Fatty acid and phospholipid metabolism
Degradation				
		56	26 (46.4)	54 (96.4)	
Protein synthesis
Electron transport, Other, Ribosomal proteins: synthesis and modification
				
	151	142 (94.0)	107 (70.9)*	
Cellular processes
Pathogenesis
				
	22	12 (54.5)	21 (95.5)	
Mobile and extrachromosomal element functions
Prophage functions, Pathogenesis
Transposon functions
				
	75	11 (14.7)	42 (56)	
	157	142 (90.4)	99 (63.1)*	
Hypothetical proteins
Conserved
Domain
No data
				
	1720	1360 (79.1)	1466 (85.2)	
	137	76 (55.5)	100 (73.0)	
	1407	919 (65.3)	1015 (72.1)	
Unknown function
Enzymes of unknown specificity
General, No data
				
	700	564 (80.6)	646 (92.3)	
	996	842 (84.5)	893 (89.7)	
All main roles
Other
				
	1288	940 (73.0)	1065 (82.7)	
*The only functional groups in which the chromosomal cpe-carrying strains carry more genes than the plasmid-borne strains.
