# Supplementary material for: Comparative Genomic Hybridization Analysis Shows Different Epidemiology of Chromosomal and Plasmid-Borne cpe-Carrying Clostridium perfringens Type A
Source: PLoS One. 2012 Oct 19;7(10):e46162. doi: 10.1371/journal.pone.0046162 (PMC3477167; doi:10.1371/journal.pone.0046162)
Supplement: Table S3 — Utilization of myo-inositole, ethanolamine, and cellobiose of chromosomal and plasmid-borne cpe-carrying C. perfringens strains. (RTF) [file pone.0046162.s004.rtf]

Table S3. Utilization of myo-inositole, ethanolamine, and cellobiose of chromosomal and plasmid-borne cpe-carrying C. perfringens strains.
	
Strain	cpe-location
	Carriage of differentiating operons or gene clustersa		Utilization of substrates as the only source of energyb	
		myo-inositole
	Ethanolamine	Cellobiose		myo-inositole	Ethanolamine	Cellobiose	
C545
	C	-	-	+		-	-	+	
CPLi7B3
	C	-	-	+		-	NT	+	
C776
	C	-	-	+		-	-	+	
20/85
	C	-	-	+		-	-	+	
26/86
	C	-	-	+		-	-	+	
C645
	C	-	-	+		-	-	+	
1293/86
	C	-	-	+		-	-	+	
C797/07
	C	-	-	+		-	-	+	
318/86
	C	-	-	+		-	NT	+	
C746
	C	-	-	-		-	-	-	
AAD1900a
	P	+	+	-		+	+	-	
CPi75-4
	P	+	+	-		+	NT	-	
CPi75K-4
	P	+	+	-		+	NT	-	
CPM77e
	P	+	-	-		+	-	-	
CPM77b
	P	+	-	-		+	-	-	
CPLi2-1
	P	+	+	-		+	+	+c	
C269
	P	+	+	-		+	+	-	
721/84
	P	+	+	-		+	NT	-	
1533/86
	P	+	+	-		NT	NT	-	
AAD1863c
	P	+	+	-		+	+	-	
SIDS14os
	P	+	-	-		+	-	+c	
C774
	P	+	-	-		+	NT	+c	
a +, the operon/gene cluster was present; -, the operon/gene cluster was absent
b +, the strain utilized the substrate; -, the strain did not utilize the substrate; NT, not tested 
c , the strain utilized cellobiose, but did not carry the gene cluster predicted to encode cellobiose utilization
