# Supplementary material for: Comparative Genomic Hybridization Analysis Shows Different Epidemiology of Chromosomal and Plasmid-Borne cpe-Carrying Clostridium perfringens Type A
Source: PLoS One. 2012 Oct 19;7(10):e46162. doi: 10.1371/journal.pone.0046162 (PMC3477167; doi:10.1371/journal.pone.0046162)
Supplement: Table S4 — The presence (+) and absence (−) of operons and gene clusters encoding the metabolic traits differentiating between the chromosomal and plasmid-borne cpe-carrying C. perfringens strains. (RTF) [file pone.0046162.s005.rtf]

Table S4. The presence (+) and absence (-) of operons and gene clusters encoding metabolic traits differentiating between the chromosomal and plasmid-borne cpe-carrying C. perfringens strains.
Location of the cpe	An operon or a gene cluster differentiating C. perfringens strains	
					
	myo-inositol	Ethanolamine	Cellobiose	Biotin	
					
chromosomal	-	-	+	-	
plasmid-borne	+	+	-	+	
